# Synthesis and In Vitro Antimicrobial Evaluation of Photoactive Multi—Block Chalcone Conjugate Phthalimide and 1,8-Naphthalimide Novolacs

**DOI:** 10.3390/polym13111859

**Published:** 2021-06-03

**Authors:** Periyan Durairaju, Chinnasamy Umarani, Govindasami Periyasami, Perumberkandigai Adikesavan Vivekanand, Mostafizur Rahaman

**Affiliations:** 1 Department of Chemistry, Thiruvalluvar Government Arts College, Namakkal 637401, India; 2 Department of Chemistry, Government Arts College (Autonomous), Salem 636007, India; sachuutay@gmail.com; 3 Department of Chemistry, College of Science, King Saud University, Riyadh 11451, Saudi Arabia; mrahaman@ksu.edu.sa; 4 Department of Chemistry, Saveetha Engineering College, Chennai 602105, India; pavivek@rediffmail.com

**Keywords:** N-substituted phthalimide, fluorescent naphthalimide, chalcone spacer, phenol-formaldehyde resins, antimicrobial activity

## Abstract

Herein we report new multiblock chalcone conjugate phthalimide and naphthalimide functionalized copolymers with a topologically novel architecture synthesis using nucleophilic substitution and polycondensation methodology. The structures of the synthesized novolacs were elucidated on the basis of their spectroscopic analysis including FTIR, ^1^H NMR, and ^13^C NMR spectroscopy. Further, the number-average and weight-average molecular weights of the novolac polymers were determined by gel permeation chromatography (GPC). We examined the solubility of the synthesized polymers in various organic solvents including CHCl_3_, CH_3_CN, THF, H_2_O, CH_3_OH, DMSO, and DMF and found they are insoluble in both methanol and water. The novolac polymers were evaluated for their photophysical properties and microbial activities. The investigation of the antimicrobial activities of these polymers reveals significant antimicrobial activity against the pathogens *E. coli*, *S. aureus*, *C. albicans*, and *A. niger*.

## 1. Introduction

Polymeric fluorescent compounds are an attractive research area for researchers in both academia and industries including textiles, food, cosmetics, and biomedicines. Among the various available fluorescent materials in the literature, polymeric compounds containing phthalimides and 1,8-naphthalimide analogs exhibit a wide range of applications [1,2,3]. The structural feature of –CO–N(R)–CO– and the imide ring functionality in phthalimides are responsible for the bioactivity and pharmaceutical properties of the analogs. Interestingly, due to their hydrophobic and neutral nature, phthalimides can cross biological membranes in vivo [4]. Moreover, they exhibit antioxidant and anti-inflammatory properties [5,6], have antimicrobial potential [7], and possess anthelmintic activity [8]. In 2012, Basaric et al. found that phthalimides can produce tumor necrosis factor (TNFα) to suppress tumor cell proliferation [9]. Furthermore, phthalimide polymers are widely used in biomedical and biological fields with a variety of properties including antimalarial, anti-influenza, anticancer, anti-inflammatory, and antifungal activities, apoptotic-inducing properties, and in applications such as protein sensors, n-type organic semiconductors, and electroluminescence [10,11,12,13,14].

Similarly, naphthalimide and its derivatives are a unique class of fused naphthalene compounds in which a chalcone is fused to a naphthalimide moiety. Naphthalimide derivatives possess a broad range of biological activities such as antiviral [15], antioxidant [16], antitumor [17], and antimicrobial activities [18]. In 2017, Al-Azzawi and Faiq reported a variety of new phenolic resins bearing 1,8-naphthalimide pendent groups for various applications [19]. They also act as blue-light-emitting materials because of their highly efficient fluorescence and photoluminescence, thermal and oxidative stability, and emission of polarized blue light [20]. Hence, during recent decades, remarkable advances have been made in the production of naphthalimide polymeric derivatives. For instance, allyloxy- and alkoxy-substituted 1,8-naphthalimide copolymeric materials emit bright blue fluorescence and can be used as brightening agents for polymers [21]. In 2020, Hudson et al. [22] reported that 1,8-naphthalimide-based deep-red acrylic polymers could be applied as ratiometric temperature sensors. Very recently, a ratiometric fluorescent nanoprobe based on naphthalimidefunctionalized carbon dots was developed for HeLa cellline studies [23]. Motivated by their notable fluorescent and biological characteristics, we were interested in investigating phthalamide- and naphthalimide- based fluorescent novolac analogs. In this work, we propose the synthesis and characterization of a new type of highly structured chalcone conjugated fluorescent resins bearing naphthalimide and phthalimide pendants on the novolac backbone. Further, the synthesized fluorescence novolac resins exhibited in vitro inhibitory activity against highly threatening pathogens.

## 2. Materials and Methods

All required chemicals were purchased from Sigma (Bangalore, Karnataka, India), Merck (Mumbai, Maharashtra, India), and Alfa Aesar (Melbourne, Australia). ^1^H and ^13^C NMR spectra were recorded on a Bruker 400 MHz instrument (SAIF-IITM, Chennai, India). UV–Vis absorption spectra were recorded on a Perkin Elmer LAMBDA 950 spectrophotometer (SAIF-IITM, Chennai, India), and fluorescence measurements were performed on a Spectra Max Fluorolog-3 instrument (SAIF-IITM, Chennai, India) at room temperature. Silica gel (60–230 mesh) (LOBA Chemie, India) was employed for routine column chromatography separations. Thin layer chromatography (TLC) was performed on precoated (0.25 mm) silica gel F 254 plates (E. Merck, Germany); naphthalimide derivatives were detected using a 254 nm UV lamp. Melting points were recorded on MEL-TEMP Electrothermal melting point apparatus (Electrothermal, Germany) and were uncorrected.

### 2.1. General Procedure for the Synthesis of Isoindoline-1,3-Diones 3 and 7

Isoindoline-1,3-diones 3 and 7 were prepared following a literature procedure [24]. Briefly, 4-aminoacetophenone was reacted with the appropriate acid anhydride in glacial acetic acid (50 mL). The reaction mixture was heated to boiling temperature (118 °C) for 16 h with constant stirring and monitored by TLC to determine the completion of the reaction. Then, the reaction mixture was allowed to cool to room temperature, and ice-cold water was added. The resulting solid was filtered off and dried, and the crude product was purified by recrystallization from a hot EtOH:CHCl_3_ (1:1) mixture.

### 2.2. General Procedure for the Condensation Reaction to Synthesize **4a**–**c** and **8a**–**d**

An equimolar mixture of the appropriate aromatic aldehyde and isoindoline-1,3-dione 3 or 7 was stirred with 10–20 mL of ethanol, and 10 mL of a 40% aqueous KOH solution was added at room temperature. The reaction mixture was stirred overnight, and the completion of the reaction was determined by TLC. The reaction mixture was poured into ice-cold water and acidified with dilute HCl to precipitate the crude product, which was then recrystallized with hot ethanol.

### 2.3. General Procedure for the Synthesis of Compounds **5a**–**c** and **9a**–**d**

An equimolar mixture of chalcone **4a** and formaldehyde was placed in a 250 mL round bottom flask fitted with a mechanical stirrer and a condenser to allow melting at 60 °C with constant stirring under N_2_ atmosphere. Then, 5 mL of TEA was added dropwise, and the reaction mixture was allowed to reflux for a further 6–7 h under N_2_ atmosphere. After TLC indicated consumption of the reactants, the reaction mixture was allowed to cool down. Then, the excess of TEA was removed by washing with diethylether (3 × 15 mL), and the solid mass was poured into hot ethanol and dried overnight under vacuum in a hot oven at 50 °C.

### 2.4. Determination of Molecular Weight

The most significant parameter in polymer characterization is the determination of molecular weight. In the current study, we found the molecular weights of the novalac polymers using gas permutation chromatography (GPC), a reliable and systematic technique. The number-average molecular weights (Mn) and the weight-average molecular weights (Mw) of the polymer samples were determined using a Waters 510 HPLC pump (Waters, Hayward, NJ, USA) equipped with a Waters 410 Differential Refractometer instrument (Waters, Hayward, NJ, USA) at an operating wavelength (λ) of 930 nm. Gel permeation chromatography (GPC) consisted of three sets of Waters Styragel columns, viz., HR1, HR2, and HR3, having a size of 7.8 mm in diameter and 300 mm in length. Initially, the instrument calibration techniques were done according to the standard method with poly(styrene) standard and the experiment was carried out at 40 ± 1 °C using HPLC grade acetonitrile (CH_3_CN) as the mobile phase at a flow rate of 1.0 mL/min.

### 2.5. Spectrophotometric Studies

#### 2.5.1. FourierTransform Infrared Spectroscopy

The Fouriertransform infrared spectroscopy (FTIR, Shimadzu, Japan) spectra were recorded in a Shimadzu FTIR 8400 spectrophotometer instrument using a KBr disc back as a reference. Spectra were acquired between 4000 and 400 cm^−1^, accumulating up to 45 spectra with a resolution of 2 cm^−1^.

#### 2.5.2. Absorption and Emission Spectral Studies

The UV–Vis double beam UVD-3500 Labomed Inc. (Los Angeles, CA, USA) instrument was used to record absorption spectra at room temperature, and the emission spectra were recorded in a PerkinElmer Luminescence PE 45 instrument. Spectroscopic grade acetonitrile was used as a solvent to record the emission spectra, and the concentration of 1 × 10^−6^ M synthesized novolac solutions was prepared by sonication for 10–15 min as it has poor solubility. The slit width was 5 nm at input and output pathways and the path length of the cuvette was 10 mm.

### 2.6. Antimicrobial Studies

Antimicrobial activities of the synthesized phthalimide and naphthalimide novolacs were used to screen primary biological studies. All the synthesized compounds **5a**–**c** and **9a**–**d** were subjected to antimicrobial activity against common pathogens such as Gram-negative bacteria (*Escherichia coli* MTCC 25922), Gram-positive bacteria (*Staphylococcus aureus* MTCC 25923), pathogenic yeast (*Candida albicans* MTCC 282), and a fungus (*Aspergillus niger* MTCC 227), which are usually found in vegetables and fruits. The agar diffusion method was used for the determination of antimicrobial activities against the above-mentioned microorganisms by standard methodology [25]. All the cultures were maintained in the standard laboratory conditions. Wells were made with a cork borer in the solid agar and loaded with 50, 75, and 100 mg/mL of the novolacs dissolved in DMSO with tetracycline and carbendazim as control for *E. coli*, *S. aureus*, *C. albicans*, and *A. niger*, respectively. Petri dishes were incubated at 35 °C for 24 h and the resulting inhibition zone diameter was measured in millimeters.

## 3. Results and Discussion

### 3.1. Synthesis

**Scheme 1:**
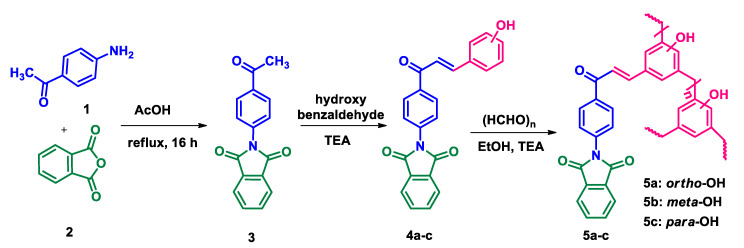
Synthesis of N-substituted phthalimide phenol analogs **5a**–**c**.

**Scheme 2:**
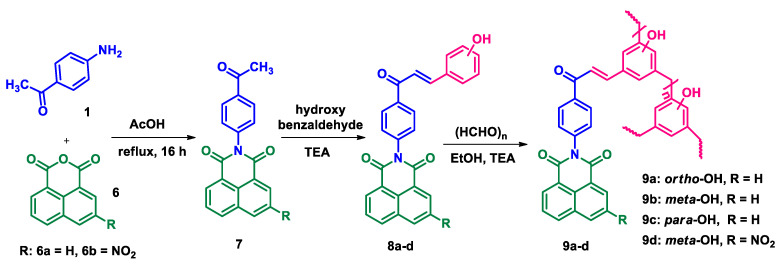
Synthesis of N-substituted naphthalimide phenol analogs **9a**–**d**.

The synthesis of phthalimide analogs **5a**–**c** and 1,8-naphthalimide analogs **9a**–**d** was performed as depicted in Scheme 1 and Scheme 2, starting from key materials 1 and 2 and 1 and 6, respectively. Initially, 2-(4-acetylphenyl)isoindoline-1,3-dione (3) and 2-(4-acetylphenyl)-5-substituted-1H-benzo[de]isoquinoline-1,3(2H)-dione (7) were synthesized according to a literature procedure [24] by the reaction of the corresponding aromatic anhydride with 4-aminoacetophenone in acetic acid under reflux. In the second step, the required chalcone was obtained by aldol condensation and subsequent dehydration of 1,3-diones 3 and 7 with various substituted aromatic aldehydes in ethanolic solution in KOH medium. The obtained crude products were dissolved with hot ethanol and recrystallized to produce chalcones **4a**–**c** and **8a**–**d**. These chalcones underwent polymerization by the reaction with formaldehyde in a ratio of 1:8 in the presence of triethylamine (TEA) in ethanolic solution at 65 °C under N_2_ atmosphere, producing phenolic-formaldehyde resins **5a**–**c** and **9a**–**d** in 50–56% yields.

**Figure 1:**
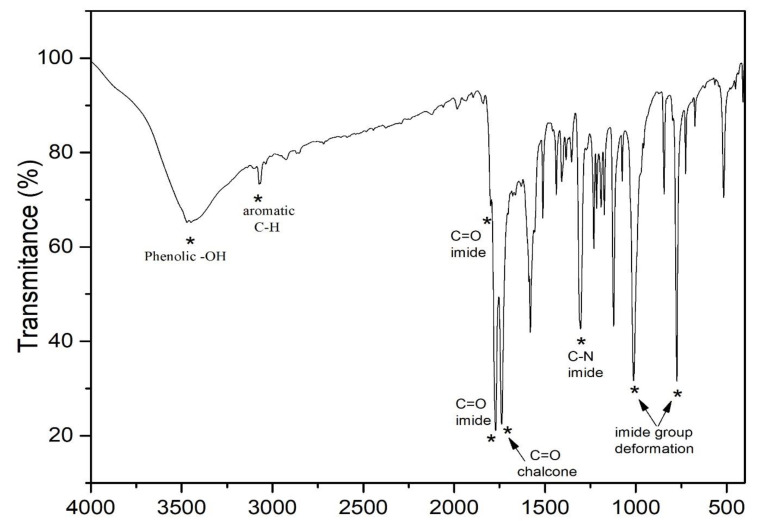
FTIR spectrum of phthalimide pendent novolac **5a**.

The structures of the newly synthesized compounds were confirmed by spectroscopic analysis. Thus, the IR spectra of **5a**–**c** and **9a**–**d** show two strong absorption peaks between 1777 and 1660 cm^−1^, which revealed the presence of two carbonyl groups. The absorption peak at 1402 cm^−1^ (imide), together with the obvious bands around 2920 cm^−1^, are attributed to the amide group, which further supports the proposed structures. A representative FTIR spectrum of phthalimide pendent novolac **5a** is shown in Figure 1. The remarkable signatures around 1790 cm^−1^ and 1769 cm^−1^ denote the asymmetrical and symmetrical stretching frequencies of imide carbonyl groups, respectively. The intense band of chalcone carbonyl carbon symmetrical stretching absorption appeared at 1727 cm^−1^. An absorption band that appeared around 1306 cm^−1^ corresponds to C–N stretching and the sharp absorption bands that appeared around 1012 and 771 cm^−1^ are due to imide ring deformation. The aromatic C–H stretching frequency has been observed at 3071 cm^−1^ and the absorption at 3460 cm^−1^ represents the presence of a phenolic OH group in the novolac polymer.

**Figure 2:**
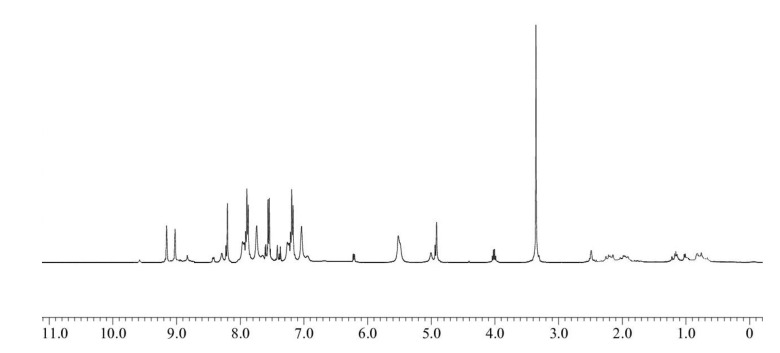
^1^H NMR spectrum of phthalimide pendent novolac **5a**.

All the newly synthesized compounds structures were determined based on the standard literature procedure [26,27]. As a representative example, the ^1^H NMR spectrum of compound **5a** shows a characteristic singlet at δ 5.46 ppm, which is due to the presence of phenolic OH and a distorted doublet at δ 3.19 ppm corresponding to the newly generated methylene group between the phenolic rings. The aromatic protons are observed between δ 7.19 and 8.22 ppm (Figure 2). The ^13^C NMR of compound **5a** shows a characteristic peak at δ 29.71 ppm that can be assigned to methylene carbons. The downfield peaks at 158.30 and 186.20 ppm confirm the presence of 1,3-diketonyl and α,β-unsaturated carbonyl carbons in compound **5a**, respectively. The structures of the synthesized compounds **5a**–**c** and **9a**–**d** are presented in Table 1, and complete NMR data can be found in the supplementary materials (S1).

### 3.2. Average Molecular Weight Analysis

**Figure 3:**
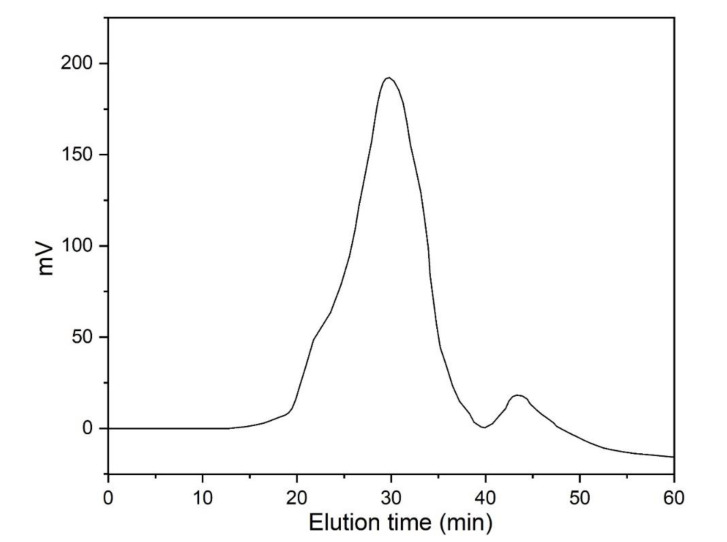
Molecular weight distribution chromatogram of novolac **5a** from GPC.

The average molecular weights (M_n_ and M_w_) and polydispersity index (Ð = M_w_/M_n_) of the synthesized polymers were calculated and the results are presented in Table 2. The chromatogram of novolac **5a** is presented in Figure 3.

The average molecular weight of phthalimide novolacs**5a**–**c** (M_n_ = 15,169–16,734, M_w_ = 46,512–52,110, and Ð = 2.82–3.43) was found to be lower than 1,8-naphthalimide novolacs **9a**–**d** (M_n_ = 12,056–18,599, M_w_ = 50,218–60,327, and Ð = 2.70–4.41). The obtained high Ð values (Ð > 3.00) of the synthesized polymers are due to fragmentation and re-combination of the monomers. Further, we attribute the high Ð values of the polymers to their branched structures. In addition, all other polymers have Ð values > 2.00 (Ð = 2.70, 2.82, and 2.91), which indicates the presence of a hyper-branching chain in the synthesized polymers. The naphthalimide analog **9d** showed the lowest Ð value (M_n_ = 9514, M_w_ = 18,326, and Ð = 1.92) compared to the other analogs due to the substituted electron-withdrawing nitro group in the naphthalimide ring.

### 3.3. Absorbance Spectra

**Figure 4:**
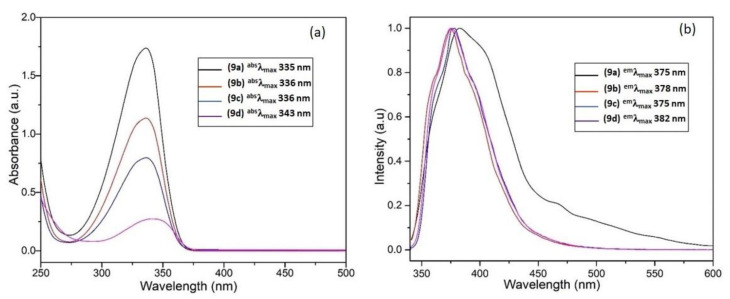
(**a**) UV–visible spectra of **9a**–**d** in acetonitrile solution; (**b**) normalized fluorescence spectra of **9a**–**d** at a concentration of 1 × 10^−6^ M in acetonitrile solution.

The UV–visible absorption spectra of naphthalimide analogs **9a**–**d** recorded in acetonitrile are shown in Figure 4a. It is obvious that the photochemical properties of the 1,8-naphthalimide core are influenced by the substituents present in the aromatic ring [28]. All the synthesized naphthalimide derivatives were UV active, with maximum absorption wavelengths (^abs^λ_max_) in the region 336–343 nm, and the acetonitrile solutions were colorless to the naked eye. The observed blue shift of the absorbance of 1,8-naphthalimide is due to the presence of the chalcone. In general, the n–π* transition of chalcone compounds is stronger than their π–π* transition owing to the presence of an extended π–conjugation system in the structure. Xiao et al. [29] reported the synthesis and characterization of 4-(2-methoxyethoxy)-N-butyl-1,8-naphthalimide (MEBN), and they found that MEBN had a ^abs^λ_max_ value at 355 nm, whereas that of 4-methoxy-N-butyl-1,8-naphthalimide was 350 nm, which was attributed to the presence of the more strongly electron-donating methoxy-ethoxy group compared to the methoxy group. The fluorescent molecules based on 1,8-naphthalimide thio and amino derivatives exhibit maximum excitation wavelengths ranging between 390 and 398 nm, which agrees well with literature reports [30].

### 3.4. Fluorescence Emission

**Figure 5:**
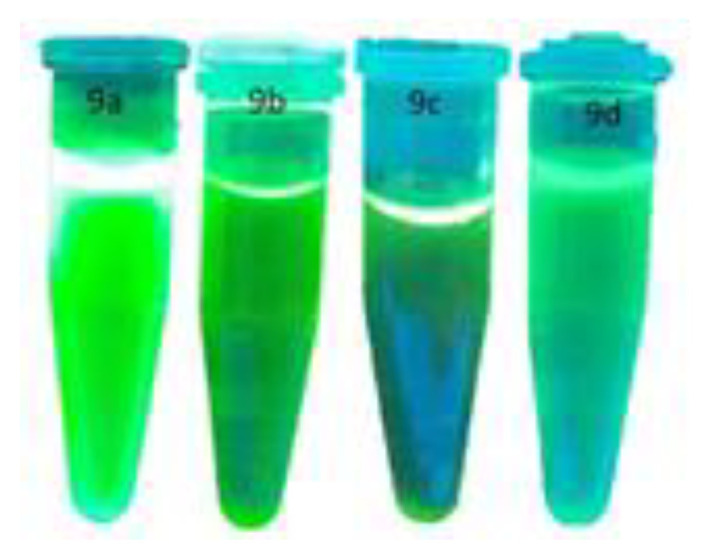
Blue light emission of **9a**–**d** under 365 nm UV irradiation.

The excited state behavior of naphthalimide analogs **9a**–**d** was evaluated by recording their fluorescent emission spectra in acetonitrile solutions (^em^λ_max_ = 375–382 nm). As shown in Figure 4b, all the naphthalimide analogs showed a similar range of emission, which is slightly red-shifted to ^em^λ_max_ = 382 nm in the case of compound **9d**. The spectrum of this compound also exhibits a shoulder at ^em^λ_max_ = 411 nm, which is due to the presence of the electron-withdrawing NO_2_ group in the 5-position of the naphthalimide ring, reducing the π–electron conjugation between the chalcone spacer and the naphthalimide aromatic ring. Table 3 summarizes the photophysical properties of naphthalimides **9a**–**d**. In addition, Figure 5 displays the fluorescence emissions of these compounds upon exposure to UV light.

As reported by Xiao et al., the fluorescence emission of MEBN was sensitive to solvent polarity, and the corresponding bands were gradually shifted to lower energies with increasing solvent polarity [29]. The effect of the polarity of the medium on the photoluminescent emission maximum was more pronounced than on the absorption maximum. The long wavelength of fluorescence emission observed in more polar solvents is due to an intramolecular charge-transfer transition. In this context, Saito et al. [30] designed three fluorescent probes integrated in a molecular system, in which the introduction of amines as receptors enabled the photo-induced electron transfer process.

### 3.5. Antimicrobial Activity

#### 3.5.1. In Vitro Antibacterial Activities

The agar well diffusion method was used to determine the antibacterial activities of the synthesized imide compounds **5a**–**c** and **9a**–**d**. The bacterial strains used in these studies were *S. aureus* MTCC 25923 and *E. coli* MTCC 25922, and tetracycline was used as a positive control. Compound **9b** was effective in controlling both pathogens with a large zone of inhibition at a concentration of 50, 75, and 100μg/mL. However, interestingly, the zone of inhibition was almost the same as that of the positive control at the higher concentration (100 μg/mL). Moreover, **5c**, **9c**, and **5b** showed antibacterial effects for both strains, with zones of inhibitions of 10–23 mm, 9–18 mm, and 8–18 mm, respectively (Table 2).

#### 3.5.2. In Vitro Antifungal Activity

**Figure 6:**
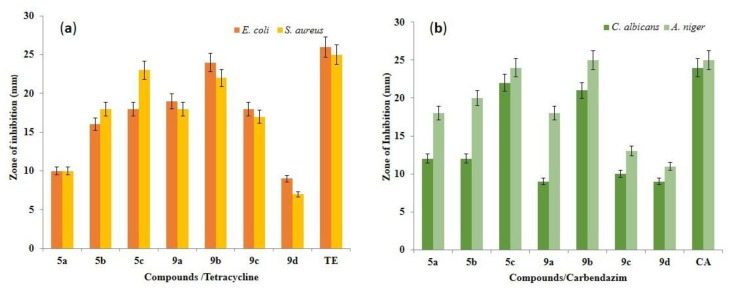
Antimicrobial efficiency of compounds **5a**–**c** and **9a**–**d**. (**a**) Zone of inhibition of tested bacteria, i.e., *E. coli* MTCC 25922 and *S. aureus* MTCC 25923. (**b**) Zone of inhibition of tested fungi, i.e., *C. albicans* MTCC 282 and *A. niger* MTCC 227. Results are expressed as mean ± SD for *n* = 3. Bars with the same letter are not significantly different at *p* > 0.05.

**Figure 7:**
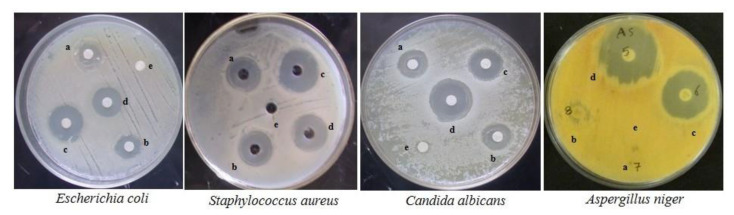
Antimicrobial activity of compound **9b** against common bacterial pathogens. a: 50 µL, b: 75 µL, c: 100 µL, d: positive control, e: negative control.

The antifungal activity of compounds **5a**–**c** and **9a**–**d** was evaluated against two fungal strains, viz., *A. niger* MTCC 227 and *C. albicans* MTCC 282, at various concentrations (μg/mL) using the disk diffusion technique and the inhibition data are presented in Table 4. Compound **9b** could effectively control both fungal pathogens with zone of inhibition values of 11–28 mm at concentrations of 50–100 μg/mL. The polymer **5c** showed a moderate zone of inhibition between 11 and 26 mm, and **5b** and **9c** exhibited efficient inhibition ranges compared with carbendazim as a positive control. Figure 6 displays a representative graph summarizing these results and the zone of inhibition of high-efficacy polymer **9b** is presented in Figure 7.

## 4. Conclusions

The synthesis of new multiblock chalcone conjugate phthalimide and naphthalimide functionalized copolymers with a topologically novel architecture synthesis using nucleophilic substitution and polycondensation methodology have been successfully explored. We have synthesized phthalimide- and naphthalimide-containing novolac analogs conjugated with a chalcone spacer via sequential nucleophilic substitution and condensation reaction in a triethylamine base. Spectroscopic analysis including FTIR, 1H NMR, and 13C NMR spectroscopy confirms the structures of the synthesized novolacs. The synthesized multi–block novolacs are photoactive; notably, 1,8-naphthalimide pendant organic functional group novolacs showed interesting optical activity compared to the other phthalimide pendent functionalized novolacs. All the 1,8-naphthalimide derivatives showed UV absorption and emission.

Employing gel permeation chromatography (GPC), the number- and weight-average molecular weights of the novolac polymers were determined. On examining the solubility of synthesized polymers in various organic solvents including CHCl_3_, CH_3_CN, THF, H_2_O, CH_3_OH, DMSO, DMF, etc., these novolac polymers were insoluble in methanol and water. Photophysical properties and microbial activities of the synthesized novolac polymers were evaluated. The investigation of the antimicrobial activities of these polymers reveals significant antimicrobial activity against the pathogens *E. coli*, *S. aureus*, *C. albicans*, and *A. niger*. The in vitro antibacterial activities against *E. coli* and *S. aureus* and the in vitro antifungal activities against *C. albicans* and *A. niger* were evaluated for the novolacs using the MIC method. The antimicrobial efficacy of all the synthesized novolacs showed interesting results for all the tested pathogens. The MIC values of all synthesized compounds are found to be effective compared to the standard drugs tetracycline and carbendazim. Particularly, compounds **9b** and **5c** showed their effective inhibition of both Gram-positive and Gram-negative pathogens. The present study strongly supports that the synthesized organic multi–block functionalized novolacs are optically active materials for materials science applications and also act as suitable material for a wide range of antimicrobial applications in health-associated sectors.

## Figures and Tables

**Table 1:** Phthalimide- and naphthalimide-substituted phenol-formaldehyde polymeric resins **5a**–**c** and **9a**–**d**.

S.No.	Aromatic Aldehyde	Product	Yield (%)
1.	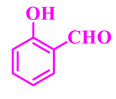	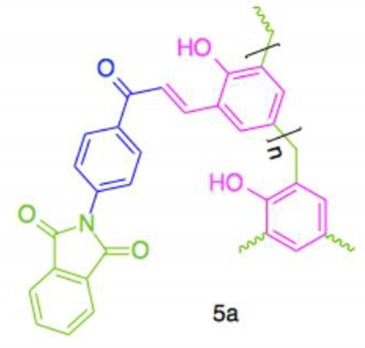	56
2.	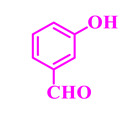	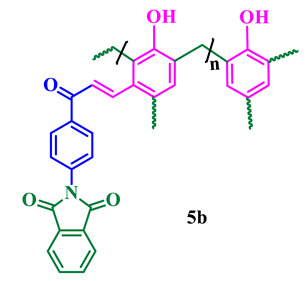	54
3.	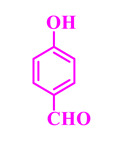	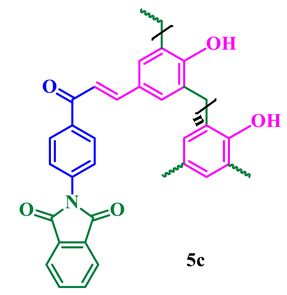	57
4.	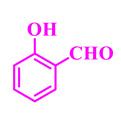	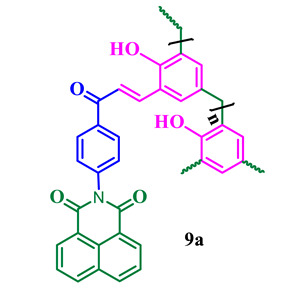	50
5.	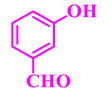	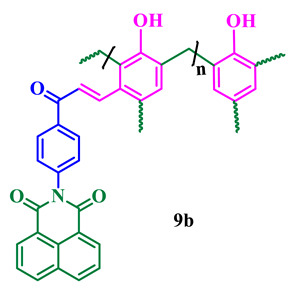	52
6.	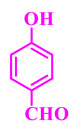	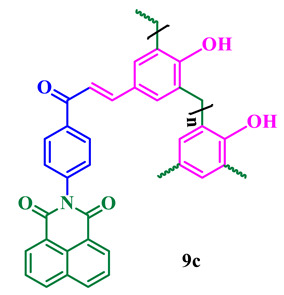	56
7.	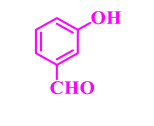	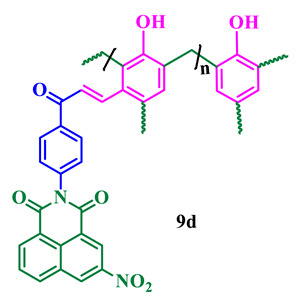	26

**Table 2:** Molecular weight M_n_, M_w_, and Ð of the synthesis novolac polymers **5a**–**c** and **9a**–**d**. (Results are expressed as rounded to the third significant number).

Polymer	Molecular Weight		
	M_n_	M_w_	Ð
**5a**	16,700	47,200	2.8
**5b**	15,169	52,100	3.4
**5c**	15,900	46,500	2.9
**9a**	12,100	53,160	4.4
**9b**	14,300	60,300	4.2
**9c**	18,600	50,200	2.7
**9d**	9500	18,300	1.9

**Table 3:** Photophysical properties of naphthalimide analogs **9a**–**d** in acetonitrile at a concentration of 1 × 10^−6^ M.

S.No.	Fluorophore	^abs^λ_max_ (nm)	^em^λ_max_ (nm)
1.	**9a**	335	375
2.	**9b**	336	378
3.	**9c**	336	375
4.	**9d**	343	382

**Table 4:** (**a**) Zone of inhibition of compounds **5a**–**c** and **9a**–**d** against bacterial pathogens. (**b**) Zone of inhibition of compounds **5a**–**c** and **9a**–**d** against fungal pathogens.

(a)						
Compounds and Positive Control	Zone of Inhibition (mm)/Concentrations (μg/mL)					
	*E. coli*			*S. aureus*		
	50	75	100	50	75	100
**5a**	8.00 ± 0.10	10.00 ± 0.14	10.00 ± 0.25	6.00 ± 0.05	9.00 ± 0.35	10.00 ± 0.75
**5b**	11.00 ± 0.15	13.00 ± 0.30	16.00 ± 0.15	8.00 ± 0.10	9.00 ± 0.22	18.00 ± 1.10
**5c**	10.00 ± 0.20	15.00 ± 0.18	18.00 ± 0.09	11.00 ± 0.80	16.00 ± 0.17	23.00 ± 0.90
**9a**	10.00 ± 0.18	12.00 ± 0.09	19.00 ± 0.45	9.00 ± 0.20	13.00 ± 0.12	18.00 ± 1.00
**9b**	16.00 ± 0.05	20.00 ± 0.40	24.00 ± 0.70	14.00 ± 0.30	17.00 ± 0.25	22.00 ± 1.20
**9c**	11.00 ± 1.05	14.00 ± 0.28	18.00 ± 0.90	9.00 ± 0.05	11.00 ± 0.20	17.00 ± 0.65
**9d**	NZI ^@^	7.00 ± 0.65	9.00 ± 0.15	NZI	6.00 ± 0.08	7.00 ± 0.15
TE *	26.00 ± 1.00			25.00 ± 0.75		
**(b)**						
**Compounds and Positive Control**	**Zone of Inhibition (mm)/Concentrations (μg/mL)**					
	***C. albicans***			***A. niger***		
	**50**	**75**	**100**	**50**	**75**	**100**
**5a**	6.00 ± 0.08	9.00 ± 0.08	12.00 ± 0.40	9.00 ± 0.10	13.00 ± 0.15	18.00 ± 0.75
**5b**	9.00 ± 0.15	11.00 ± 0.80	12.00 ± 0.20	7.00 ± 0.10	14.00 ± 0.60	20.00 ± 0.80
**5c**	12.00 ± 0.16	16.00 ± 1.00	22.00 ± 0.35	11.00 ± 0.10	18.00 ± 0.75	24.00 ± 0.65
**9a**	NZI ^@^	7.00 ± 0.05	9.00 ± 0.45	10.00 ± 0.10	16.00 ± 0.95	18.00 ± 0.50
**9b**	11.00 ± 0.55	19.00 ± 0.95	21.00 ± 0.55	12.00 ± 0.10	19.00 ± 0.45	25.00 ± 0.35
**9c**	8.00 ± 0.07	8.00 ± 0.08	10.00 ± 0.60	10.00 ± 0.10	12.00 ± 0.15	13.00 ± 0.25
**9d**	NZI	7.00 ± 0.04	9.00 ± 0.15	8.00 ± 0.10	11.00 ± 0.20	11.00 ± 0.30
CA ^$^	24.00 ± 1.25			25.00 ± 1.15		

Note: TE *: tetracycline; NZI ^@^: no zone of inhibition. CA ^$^: carbendazim; NZI ^@^: no zone of inhibition.

## Data Availability

The data presented in this study are available on request from the corresponding author.

## Supplementary Materials

The following are available online at https://www.mdpi.com/article/10.3390/polym13111859/s1, S1.
