# Supplementary material for: Synthesis and In Vitro Antimicrobial Evaluation of Photoactive Multi—Block Chalcone Conjugate Phthalimide and 1,8-Naphthalimide Novolacs
Source: Polymers (Basel). 2021 Jun 3;13(11):1859. doi: 10.3390/polym13111859 (PMC8199857; doi:10.3390/polym13111859)
Supplement: Supplementary file 1 [file polymers-13-01859-s001.zip › polymers-1209168-supplementary.pdf]

Supporting information#

Synthesis and In Vitro Antimicrobial Evaluation of Photoactive Multi–Block Chalcone Conjugate Phthalimide and 1,8-Naphthalimide Novolacs

Periyan Durairaju[1,2,*], Chinnasamy Umarani [2], Govindasami Periyasami [3,*], Perumberkandigai Adikesavan Vivekanand [4] and Mostafizur Rahaman [3]

[1] Department of Chemistry, Thiruvalluvar Government Arts College, Rasipuram, 637401, India
[2] Department of Chemistry, Government Arts College (Autonomous), Salem 636007, India; sachuutay@gmail.com
[3] Department of Chemistry, College of Science, King Saud University, Riyadh 11451, Saudi Arabia; mrahaman@ksu.edu.sa
[4] Department of Chemistry, Saveetha Engineering College, Chennai 602105, India; pavivek@rediffmail.com
* Correspondence: rajanorgyeschem@gmail.com (P.D.); pkandhan@ksu.edu.sa (G.P.)

General considerations

All required chemicals were purchased from Sigma, Merck and Alfa Aesar. ^{1}H and ^{13}C NMR spectra were recorded on a Bruker 400 MHz instrument. UV–vis absorption spectra were recorded on a Perkin Elmer LAMBDA 950 spectrophotometer, and fluorescence measurements were performed on a Spectra Max Fluorolog-3 instrument (SAIF-IITM, Chennai) at room temperature. Silica gel (60–230 mesh) was employed for routine column chromatography separations. Thin layer chromatography (TLC) was performed on precoated (0.25 mm) silica gel F 254 plates (E. Merck, India); naphthalicimide derivatives were detected using a 254 nm UV lamp. Melting points were recorded on MEL-TEMP Electrothermal melting point apparatus and were uncorrected.

1. General procedure for the condensation reaction to synthesize 4a–c and 8a–d

An equimolar mixture of the appropriate aromatic aldehyde and isoindoline-1,3-dione **3** or **7** was stirred with 10–20 mL of ethanol, and 10 mL of a 40% aqueous KOH solution was added at room temperature. The reaction mixture was stirred overnight, and the completion of the reaction was determined by TLC. The reaction mixture was poured into ice-cold water and acidified with dilute HCl to precipitate the crude product, which was then recrystallized with hot ethanol.

1.1. Compound 4a. Pale yellow solid; yield: 82%; ^{1}H NMR (400 MHz, $CDCl_3$): δ 8.92 (1H, d, *J* = 11.1 Hz), 7.92–7.72 (8H, m, ArH), 7.66 (1H, d, *J* = 11.1 Hz), 7.60 (2H, dis d), 7.50 (2H, dis d), 5.40 (1H, br s, OH) ppm; ^{13}C NMR (100 MHz, $CDCl_3$): δ 189.0, 169.0, 167.6, 152.4, 142.6, 140.5, 137.5, 137.1, 136.2, 134.8, 132.1, 128.8, 128.3, 126.4, 125.7, 125.0, 124.5, 123.3, 123.1, 122.3, 121.8, 121.1, 120.7, 120.3, 118.9 ppm; FTIR (KBr): ν_{max} 3300, 1720, 1635, 1214 cm^{-1}.

1.2. Compound 4b. Pale yellow solid; yield: 86%; ^{1}H NMR (400 MHz, $CDCl_3$): δ 8.00 (1H, d, *J* = 11.1 Hz), 7.79–7.20 (8H, m, ArH), 7.71 (1H, d, *J* = 11.1 Hz), 7.53–6.70 (4H, m, ArH), 5.48 (1H, br s, OH) ppm; ^{13}C NMR (100 MHz, $CDCl_3$): δ 189.0, 163.6, 157.5, 140.3, 137.5, 132.8, 130.6, 130.5, 130.6, 128.1, 124.5, 123.4, 122.5, 121.0, 121.5, 121.0, 120.3, 120.0, 119.5, 115.7, 110.8 ppm; FTIR (KBr): ν_{max} 3415, 1770, 1728, 1634 cm^{-1}.

1.3. Compound 4c. Colourless solid; yield: 75%; ^{1}H NMR (400 MHz, $CDCl_3$): δ 8.12 (1H, d, *J* = 11.2 Hz), 7.80–7.21 (8H, m, ArH), 7.63 (1H, d, *J* = 11.2 Hz), 7.53 (2H, dd), 6.75 (2H, dd), 5.50 (1H, br s, OH) ppm; ^{13}C NMR (100 MHz, $CDCl_3$): δ 189.1, 163.1, 158.0, 140.5, 137.5, 132.8, 130.6, 130.4, 130.1, 128.3, 124.1, 121.6, 120.5, 110.8 ppm; FTIR (KBr): ν_{max} 3412, 1772, 1728, 1635 cm^{-1}.

1.4. Compound 8a. Yellow solid; yield: 85%; ^{1}H NMR (400 MHz, $CDCl_3$^{1}H NMR (400 MHz, $CDCl_3$): δ 8.83–8.35 (6H, m), 8.23 (1H, d, *J* = 11.0), 8.11–7.94 (4H, m), 7.88 (2H, dd), 7.66 (1H, d, *J* = 11.0 Hz), 7.61 (2H, dd), 5.86 (1H, br s, OH) ppm; ^{13}C NMR (100 MHz, $CDCl_3$): 196.12, 170.12, 160.66, 140.15, 138.9, 136.2, 135.2, 135.0, 134.1, 132.5, 131.2, 130.8, 130.3, 130.0, 126.3, 127.12, 125.5, 125.1 ppm; FTIR (KBr): ν_{max} 3320, 1726, 1718, 1635 cm^{-1}.

1.5. Compound 8b. Pale yellow solid; yield: 82%; ^{1}H NMR (400 MHz, $CDCl_3$): δ 8.81-8.35 (6H, m), 8.11 (1H, d, *J* = 11.0), 7.89 (2H, dd), 7.79–7.00 (4H, m), 7.60 (1H, d, *J* = 11.0 Hz), 7.48 (2H, dd), 5.50 (1H, br s, OH) ppm; ^{13}C NMR (100 MHz, $CDCl_3$): 189.8, 159.1, 158.9, 146.3, 138.8, 137.3, 136.6, 135.3, 134.7, 133.6, 133.0, 132.7, 132.1, 131.4, 130.2, 129.5, 128.7, 128.5, 126.8, 125.0, 124.2, 123.2, 119.6, 115.2 ppm; FTIR (KBr): ν_{max} 3421, 1720, 1713, 1638 cm^{-1}.

1.6. Compound 8c. Yellow solid; yield: 56%; ^{1}H NMR (400 MHz, $CDCl_3$): δ 8.83-8.80 (6H, m), 8.23 (1H, d, *J* = 11.0), 7.86–7.71 (4H, m), 7.58 (1H, d, *J* = 11.0 Hz), 7.32 (2H, dd), 6.99 (2H, dd), 5.53 (1H, br s, OH) ppm; ^{13}C NMR (100 MHz, $CDCl_3$): 183.2, 165.0, 159.1, 143.8, 136.8, 135.3, 135.1, 134.5, 133.0, 132.6, 132.2, 131.0, 130.8, 129.7, 127.1, 126.3, 125.0, 123.4, 115.3 ppm; FTIR (KBr): ν_{max} 3410, 1726, 1718, 1635 cm^{-1}.

1.7. Compound 8d. Pale yellow solid; yield: 82%; ^{1}H NMR (400 MHz, $CDCl_3$): δ 9.13 (1H, s, ArH), 8.78 (1H, s, ArH), 8.61–8.52 (3H, m), 8.21 (1H, d, *J* = 11.5 Hz), 7.68 (2H, dd), 7.82–7.18 (4H, m), 7.55 (1H, d, *J* = 11.4 Hz), 7.06 (2H, dd), 5.95 (1H, br s, OH) ppm; ^{13}C NMR (100 MHz, $CDCl_3$): 196.1, 158.6, 158.5, 148.3, 140.6, 136.8, 136.3, 135.6, 135.3, 134.1, 133.2, 133.1, 132.5, 132.0, 131.8, 131.2, 128.5, 128.1, 127.3, 125.8, 125.3, 123.2, 122.7, 118.4, 115.0 ppm; FTIR (KBr): ν_{max} 3421, 1723, 1715, 1638, 1526 cm^{-1}.

2. General procedure for the synthesis of compounds 5a–c and 9a–d

An equimolar mixture of chalcone **4a** and formaldehyde was placed in a 250 mL round bottom flask fitted with a mechanical stirrer and a condenser to allow melting at 60 °C with constant stirring under N_2 atmosphere. Then, 5 mL of TEA was added dropwise, and the reaction mixture was allowed to reflux for further 6–7 h under N_2 atmosphere. After TLC indicated consumption of the reactants, the reaction mixture was allowed to cool down. Then, the excess of TEA was removed by washing with diethylether (3 × 15 mL), and the solid mass was poured into hot ethanol and dried overnight under vacuum in a hot oven at 50°C.

2.1. Compound 5a. White solid; yield: 56%; ^{1}H NMR (400 MHz, DMSO-d_6): δ 9.11 (1H, d, alkene H), 8.22–7.19 (10H, m, ArH), 7.80 (1H, dis-d, alkene H), 5.46 (1H, br s, OH), 3.20–3.19 (2H, dis d) ppm; ^{13}C NMR (100 MHz, DMSO-d_6): δ 186.2, 158.3, 135.1, 131.9, 131.8, 131.2, 130.5, 130.1, 129.7, 129.5, 129.3, 129.2, 111.9, 29.7 ppm; FTIR (KBr): ν_{max} 3402.82, 3064.08, 2920.5, 2851.59, 1772.07, 1738.91, 1701.89, 1631.48, 1511.53, 1312.73, 1122.3, 1013.03, 775.30, 550.30 cm^{-1}.

2.2. Compound 5b. White solid; yield: 54%; ^{1}H NMR (δ, DMSO-d_6): δ 8.92 (1H, dis-d, alkene H), 8.29-7.20 (10H, m, ArH), 7.81 (1H, dis-d, alkene H), 6.64 (1H, br s, OH), 3.21-3.19 (2H, dis d) ppm; ^{13}C NMR (100 MHz, DMSO-d_6): δ 164.49, 135.34, 134.59, 133.2, 131.72, 129.51, 129.0, 127.11, 27.97 ppm; FTIR (KBr): ν_{max} 3473.47, 3343.38, 3109.01, 3075.0, 2918.0, 2499.0, 2300.13, 1910.59, 1786.68, 744.9, 1717.75, 1600.71, 611.89, 1411.86, 1384.0,1 376, 1122.42, 10934.47, 717.72, 695, 621, 567, 479 cm^{-1}.

2.3. Compound 5c. White solid; yield: 57%; ^{1}H NMR (400 MHz, DMSO-d_6): ^{1}H NMR (δ, DMSO-d_6): δ 9.20 (1H, dis-d, alkene H), 8.25-6.86 (10H, m, ArH), 7.56 (1H, dis-d, alkene H), 5.89 (1H, br s, OH), 3.22-3.20 (2H, dis d) ppm; ^{13}C NMR (100 MHz, DMSO-d_6): δ 187.01, 160.30, 154.00, 132.2, 132.22, 131.11, 129.91, 129.02, 111.10, 31.81, 32.12, 30.33, 15.0 ppm; FTIR (KBr): ν_{max} 3430.05, 3250.08, 3060.01, 2920.0, 1778.0, 1668.5, 1366.38, 1325.8, 1180.13, 962.83, 902.14, 840.01, 777.17, 548.30, 500.67, 426.01 cm^{-1}.

2.4. Compound 9a. White solid; yield: 50%; ^{1}H NMR (400 MHz, DMSO-d_6): δ 9.15 (1H, d, alkene H), 6.98–8.28 (12H, m, ArH), 7.55 (1H, d, alkene H), 5.56 (1H, bs, OH), 3.35 (s, 2H) ppm; ^{13}C NMR (100 MHz, DMSO-d_6): 173.25, 169.01, 164.14, 160.59, 139.8, 137.11, 135.3, 134,3, 133.4, 132.8, 131.16, 130.9, 130.10, 129.46, , 127.5, 126.4, 127.1, 125.2, 122.5,

188.8, 26.8, 14.16 ppm; FTIR (KBr): ν_{max} 3350.56, 3322.09, 3097.23, 3062.32, 2992.64, 1947.49, 1702.1, 1772.4, 1739.6, 1664.70, 1622.49, 1233.8, 1013.9, 77.6, 515.98 cm^{-1}.

2.5. Compound 9b. White solid; yield: 52%; ^{1}H NMR (400 MHz, DMSO-d_6): δ 9.22 (1H, d, alkene H), 7.12–8.35 (12H, m, ArH), 7.62 (1H, d, alkene H), 5.35 (1H, bs, OH), 3.76 (s, 2H) ppm; ^{13}C NMR (100 MHz, DMSO-d_6): 163.8, 163.6, 160.5, 139.4, 136.2, 132.4, 131.5, 131.0, 130.1, 129.5, 128.0, 127.5, 123.2, 121.7, 120.7, 114.9, 114.2, 55.4 ppm; FTIR (KBr): ν_{max} 3435.85, 2980.98, 2291.10, 1121.51, 1016.35, 775.20, 569.09 cm^{-1}.

2.6. Compound 9c. White solid; yield: 56%; ^{1}H NMR (400 MHz, DMSO-d_6): δ 9.00 (1H, d, alkene H), 6.76–8.05 (12H, m, ArH), 7.64 (1H, d, alkene H), 5.02 (1H, bs, OH), 3.33 (s, 2H) ppm; ^{13}C NMR (100 MHz, DMSO-d_6): 197.27, 164.13, 139.7, 137.2, 135.34, 134.60, 133.7, 132.34, 131.82, 130.38, 130.80, 130.49, 27.21, 127.12, 125.5, 122.56, 118.9, 61.30, 29.9, 26.79, 14.16 ppm; FTIR (KBr): ν_{max} 3350.56, 3322.07, 3062.32, 2920.22, 2851.88, 2592.01, 1772.38, 1702.05, 1664.70, 1233.78, 1186.80, 1122.04, 1013.08, 831.96, 775.56, 644.70, 515.97, 477.52 cm^{-1}.

2.7. Compound 9d. Pale yellow solid; yield: 26%; ^{1}H NMR (400 MHz, DMSO-d_6): δ 9.13 (1H, d, alkene H), 8.86 (2H, dis m, ArH), 7.21–8.13 (10H, m, ArH), 7.64 (1H, d, alkene H), 5.86 (1H, bs, OH), 3.10 (s, 2H) ppm; ^{13}C NMR (100 MHz, DMSO-d_6): 206.89, 121.81, 116.05, 103.76, 96.78, 52.65 ppm; FTIR (KBr): ν_{max} 3375.34, 3100.23, 2927.21, 2864.61, 1787.769, 1619.44, 1666.72, 1485.07, 1334.64, 1282.04, 1002.30, 703.21, 600.2, 470.12 cm^{-1}.
